# Supplementary material for: Strain-restricted transfer of ferromagnetic electrodes for constructing reproducibly superior-quality spintronic devices
Source: Nat Commun. 2024 Jan 29;15:865. doi: 10.1038/s41467-024-45200-7 (PMC10824775; doi:10.1038/s41467-024-45200-7)
Supplement: Supplementary file 1 — Supplementary Information [file 41467_2024_45200_MOESM1_ESM.pdf]

Supplementary Information

Strain-restricted transfer of ferromagnetic electrodes for constructing reproducibly superior-quality spintronic devices

Lidan Guo[1†], Xianrong Gu[1†], Shunhua Hu[1,2†], Wenchao Sun[3], Rui Zhang[1,4], Yang Qin[1], Ke Meng[1,2], Xiangqian Lu[5], Yayun Liu[1], Jiaxing Wang[4], Peijie Ma[4], Cheng Zhang[1], Ankang Guo[1,6], Tingting Yang[1,2], Xueli Yang[1,6], Guorui Wang[7], Yaling Liu[1], Kai Wang[8], Wenbo Mi[3], Chuang Zhang[6], Lang Jiang[6], Luqi Liu[1], Kun Zheng[4], Wei Qin[5]*, Wenjing Yan[9], Xiangnan Sun[1,2,10]*

*Correspondence to: Wei Qin (wqin@sdu.edu.cn); Xiangnan Sun (sunxn@nanoctr.cn)

†These authors contributed equally to this paper.

This file includes:

Supplementary Note 1 and Supplementary Note 2

Supplementary Fig. 1 to Supplementary Fig. 15

Supplementary References

Supplementary Note 1: Explanation and description of attenuation structure tomography in Supplementary Fig. 10.

For the evaporation of several nanometers (such as 3 nm) $Ni_{80}Fe_{20}$ on prepared $PC_{71}BM$ layer, the hot $Ni_{80}Fe_{20}$ atoms can penetrate into the $PC_{71}BM$ layer very easily, where $Ni_{80}Fe_{20}$ islands are formed to present non-dense $Ni_{80}Fe_{20}$ film at $PC_{71}BM/Ni_{80}Fe_{20}$ interface. Once applying soft plasma etching, $PC_{71}BM$ is stripped away layer by layer. As a result, a decreased absorption is obtained with continuous tomography for the structure of $PC_{71}BM/Ni_{80}Fe_{20}$ prepared by LN-cooling-method (Supplementary Fig. 10a). However, as for the structure of $PC_{71}BM/Ni_{80}Fe_{20}$ (3 nm) prepared by lamination method, absorption does not show attenuation (Supplementary Fig. 10b), meaning the lamination method can help to form high-quality $Ni_{80}Fe_{20}/PC_{71}BM$ interlayer.

Supplementary Note 2: Reason for the magnetoresistance improvement of SV prepared by polymer-assistant strain-restricted transfer technique.

The reasons for the magnetoresistance (*MR*) improvement of SV prepared by polymer-assistant strain-restricted transfer technique has been analysed as follow. For LN-cooling methods, the FM atoms penetrated into $PC_{71}BM$ will surely enhance the interaction between them (electronic structure will also change), $Ni_{80}Fe_{20}$ will transfer electrons to $PC_{71}BM$ (based on density functional theory simulation, Supplementary Fig. 11) to form nickel-iron ions in $PC_{71}BM$ layers.[1,2] As a result, nickel-iron ions and $PC_{71}BM$ can be confined together to form a complex through nickel-iron^{+}-$PC_{71}BM^{-}$ interaction. Nickel-iron ions (not ferromagnetic nickel-iron) in $PC_{71}BM$ layer could lead to a pronounced SOC effect in $PC_{71}BM$ layer to enhance spin relaxation. Thus, such large spin relaxation in $PC_{71}BM$ layer originated from LN-cooling depositing $Ni_{80}Fe_{20}$ will weaken the spin injection and transport in $PC_{71}BM$ layer. In contrast, the polymer-assistant strain-restricted transfer technique will effectively prevent the penetration of $Ni_{80}Fe_{20}$ into $PC_{71}BM$ layer, leading to a relatively weak spin relaxation and thus enhanced spin injection and spin transport, finally a larger *MR* is obtained.

Supplementary Figures

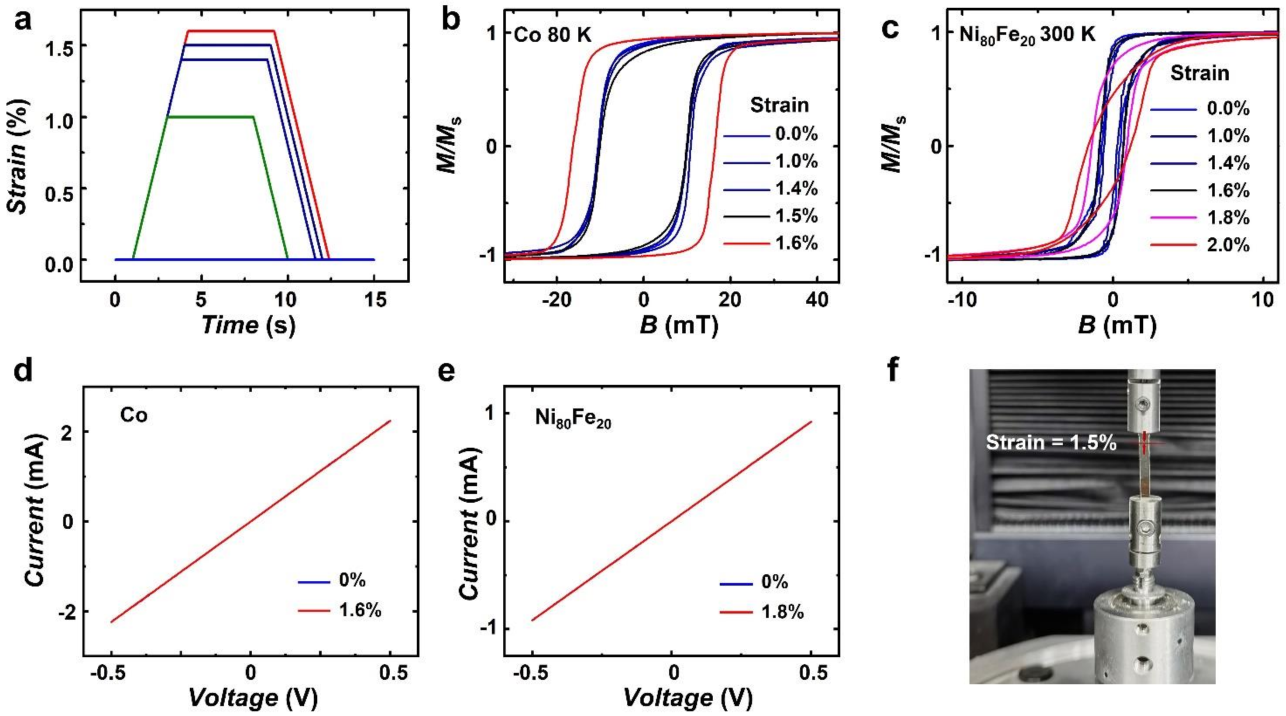

Supplementary Fig. 1

a, Setting of the strain history of FM deposited on polyethylene terephthalate (PET) substrate. **b**,**c**, Magnetic-hysteresis curves of Co at 80 K (**b**) and $Ni_{80}Fe_{20}$ at 300 K (**c**) versus strain history. **d**, Current-voltage curves of Co electrodes before (blue line) and after (red line) a strain of 1.6%. **e**, Current-voltage curves of $Ni_{80}Fe_{20}$ electrodes before (blue line) and after (red line) a strain of 1.8%. **f**, Photo of 1.5% strain of Co deposited on PET substrate.

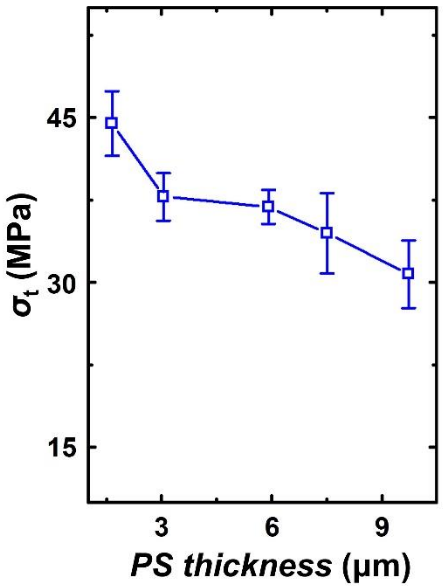

Supplementary Fig. 2

Tensile strength (σ_t) of PS versus thicknesses. Error bars represent the variation range in σ_t during the measurements.

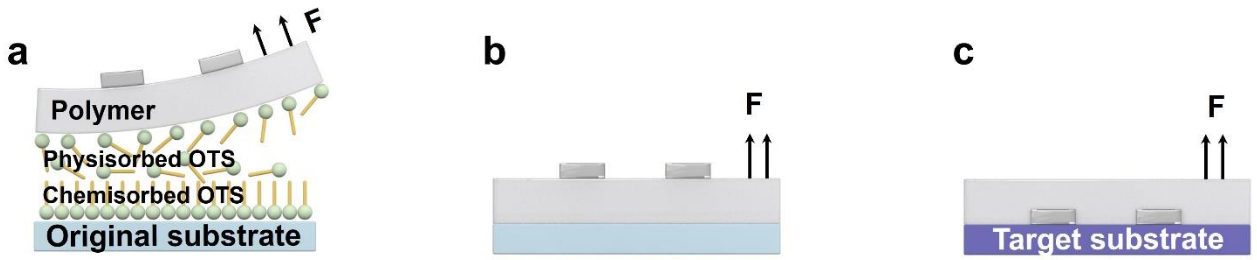

Supplementary Fig. 3

a, Schematic diagram of peeling off polymer thin film from the OTS-modified original substrate, where the peeling force is relatively small. The OTS modification contains chemical adsorption (linked to the original substrate) and physical adsorption (residual OTS), and the physical adsorption plays a crucial role on reducing the adhesion force between polymer and the original substrate. **b**, Schematic diagram of peeling off polymer thin film from the unmodified original substrate, where the peeling force is large and it is hard to peel off the polymer. **c**, Schematic diagram of peeling off polymer thin film from the targeted substrate in an already laminated sample, where the peeling force is large and it is hard to peel off the polymer.

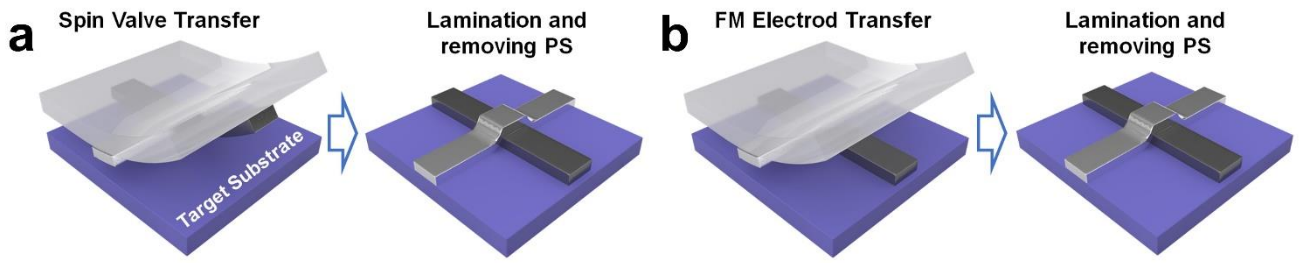

Supplementary Fig. 4

a, Sketch of transferring the whole SV and then removing the PS film. **b**, Sketch of transferring top-FM electrode to construct an SV and then removing the PS film.

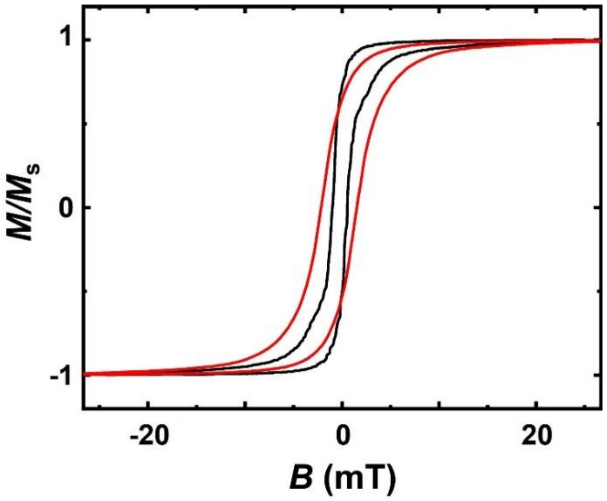

Supplementary Fig. 5

Hysteresis loops of $Al_2O_3/Ni_{80}Fe_{20}$, where the $Ni_{80}Fe_{20}$ were fabricated by strain-restricted transfer (black) and LN-cooling evaporation (red) techniques, respectively.

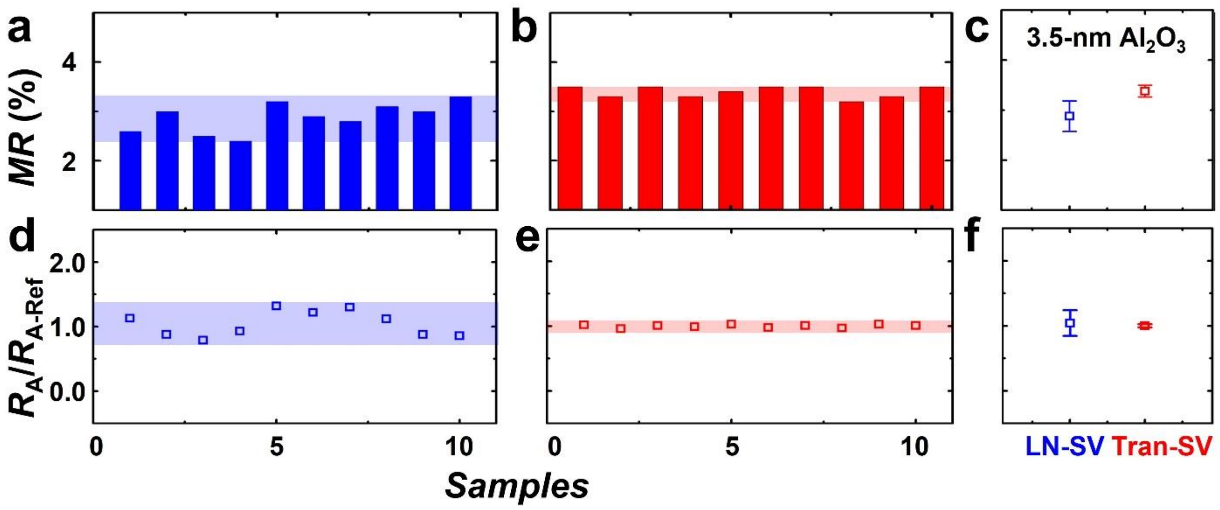

Supplementary Fig. 6

a,**b**, Comparisons of *MR* measured from randomly selected Co/Al_2O_3/$Ni_{80}Fe_{20}$ SVs prepared via (**a**) deposition (blue) and (**b**) transfer (red) processes (10 each). **c**, Statistic of *MR* from 10 devices prepared by deposition and transfer technique. **d**,**e**, Comparisons of $R_A/R_{A\text{-Ref}}$ ratio measured from randomly selected Co/Al_2O_3/$Ni_{80}Fe_{20}$ SVs prepared via (**d**) deposition (blue) and (**e**) transfer (red) processes (10 each), where R_A is the area resistance and $R_{A\text{-Ref}}$ is the mean value of R_A on the same chip. **f,** Statistic of $R_A/R_{A\text{-Ref}}$ from 10 devices prepared by deposition and transfer technique.

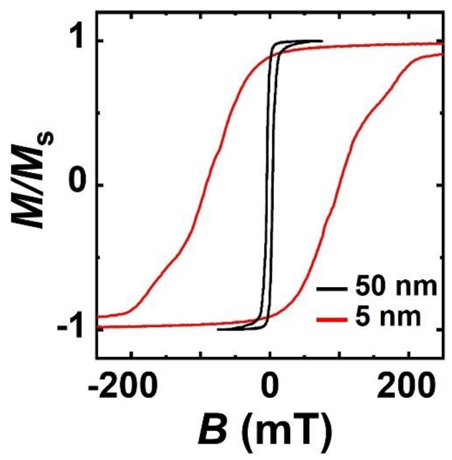

Supplementary Fig. 7

Hysteresis loops of 50-nm-thick (black line) and 5-nm-thick (red line) $Ni_{80}Fe_{20}$ onto $PC_{71}BM$ channel materials at 300K.

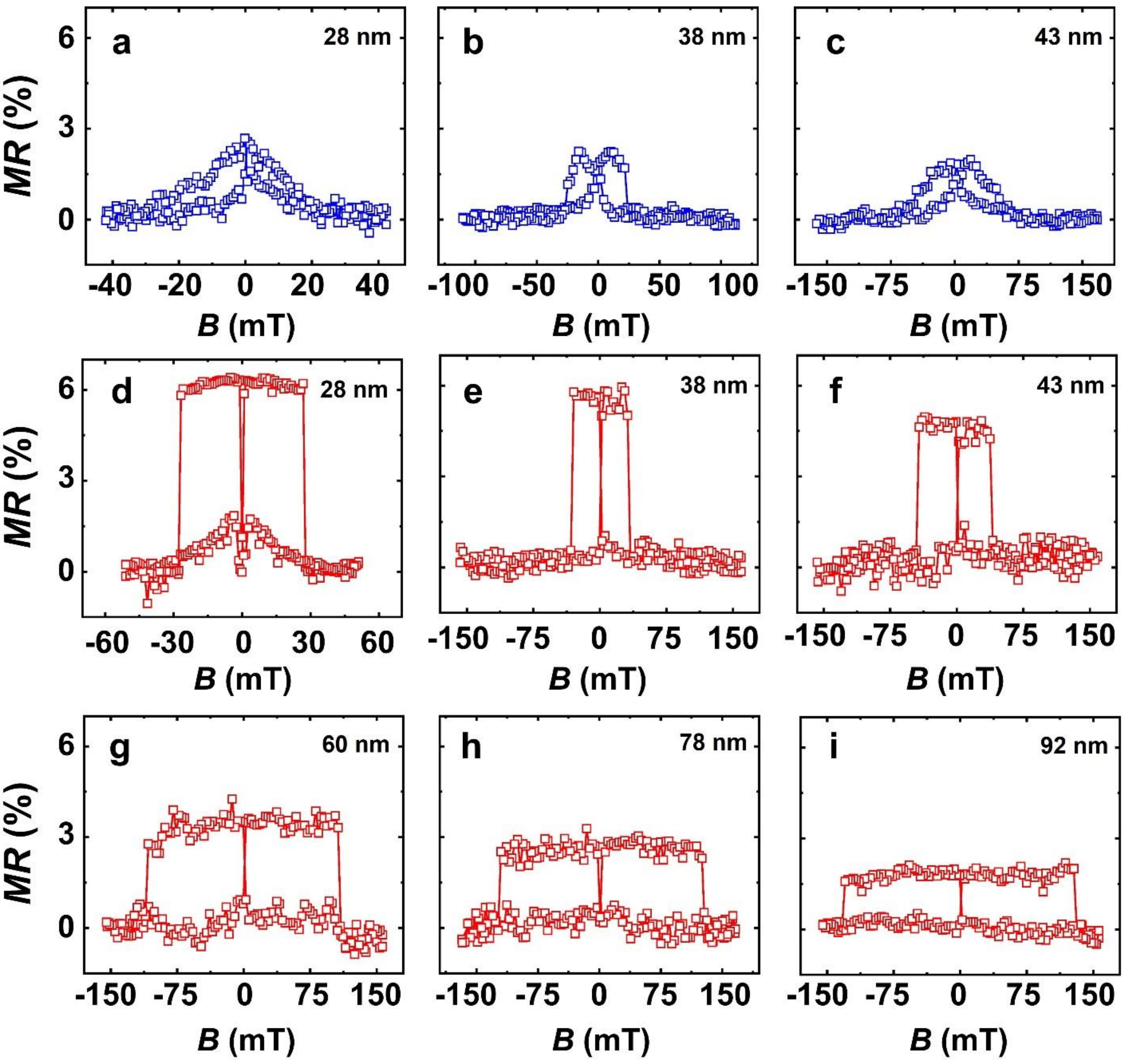

Supplementary Fig. 8

a-c, *MR* curves of $PC_{71}BM$-based SVs prepared by LN-cooling technique, where the thicknesses of $PC_{71}BM$ are 28 nm, 38 nm, 43 nm, respectively. **d-i**, *MR* curves of $PC_{71}BM$-based SVs prepared by transfer technique, where the thicknesses of $PC_{71}BM$ are 28 nm, 38 nm, 43 nm, 60 nm, 78 nm, 92 nm, respectively.

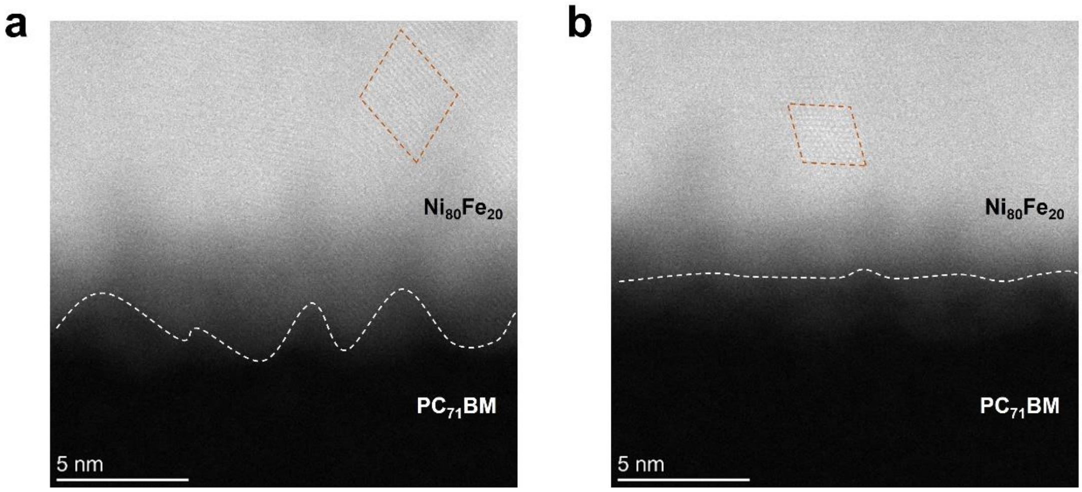

Supplementary Fig. 9

a,**b**, Cross-sectional high-resolution transmission electron microscopy (HRTEM) images of $Ni_{80}Fe_{20}/PC_{71}BM$ interfaces prepared by (**a**) LN-cooling and (**b**) transfer methods. The dashed frames of the parallelogram show the lattice fringes in $Ni_{80}Fe_{20}$ layers, and dashed curves show the interface morphologies.

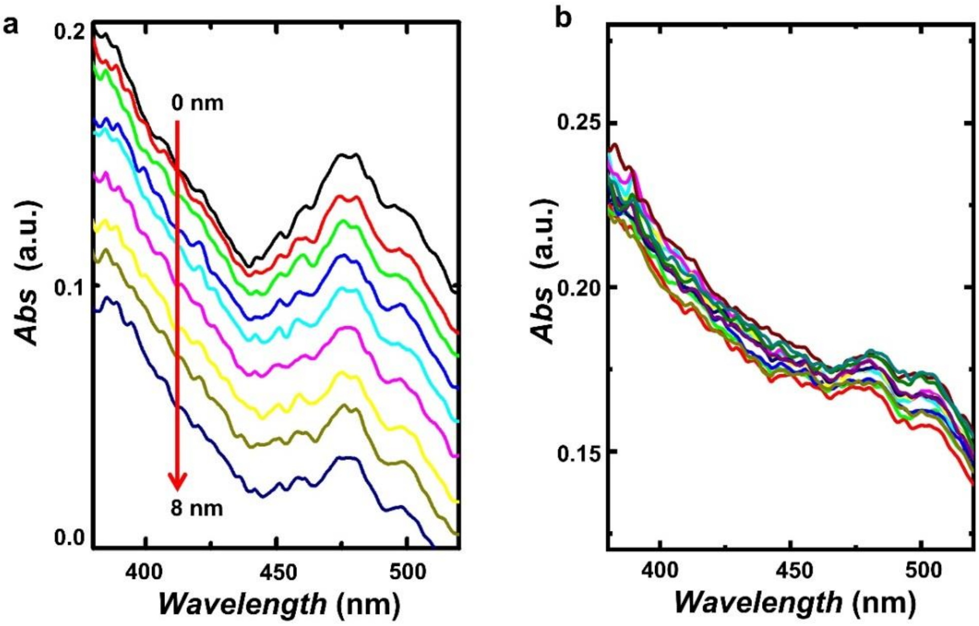

Supplementary Fig. 10

a,**b**, Attenuation structure tomography through in-situ ultraviolet-visible tomography analysis of (**a**) LN-cooling prepared sample and (**b**) transfer prepared sample, which indicates $Ni_{80}Fe_{20}$ diffusion into the $PC_{71}BM$ layer between 3~6 nm via LN-cooling preparation.

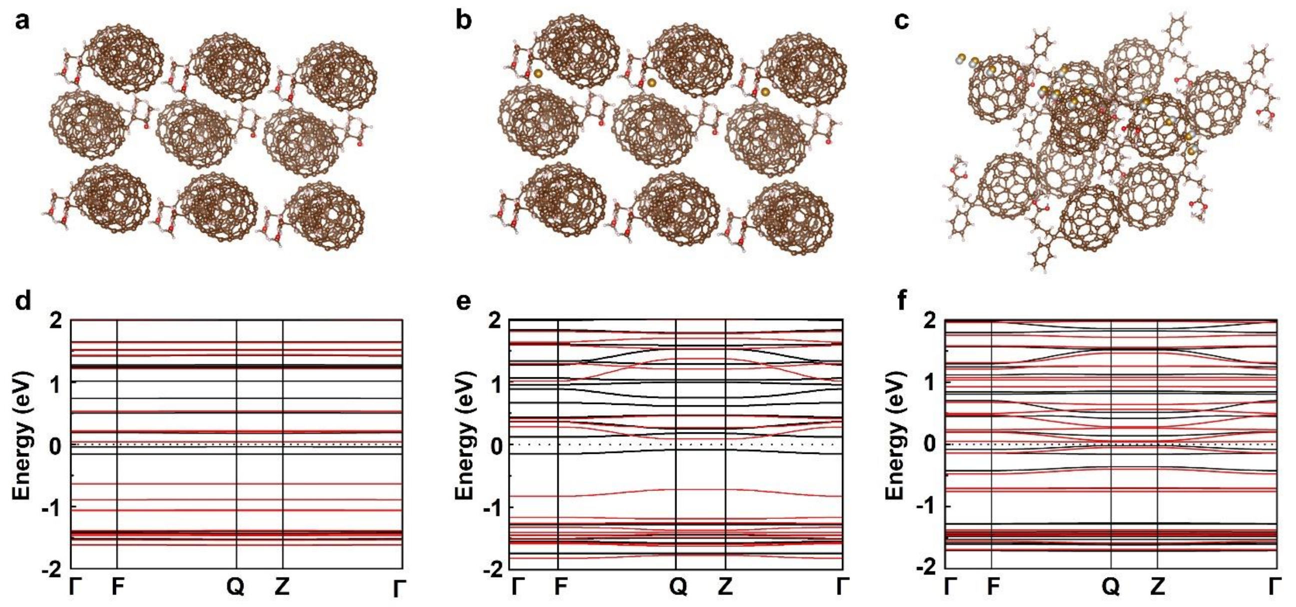

Supplementary Fig. 11

a-c, Density functional theory (DFT) calculation models of nickel and iron atoms penetrating into the $PC_{71}BM$ layer from none (**a**) to small (**b**) and large amounts (**c**), the corresponding charge transfer from $Ni_{80}Fe_{20}$ to $PC_{71}BM$ layer are zero, 0.42, 0.58 electrons. **d-f**, Energy band structure of interfacial $PC_{71}BM$ molecules affected by different degrees of $Ni_{80}Fe_{20}$ penetration in spin valves from none (representing transferred $PC_{71}BM/Ni_{80}Fe_{20}$ interface) (**d**) to small (**e**) and large amounts (representing LN-cooling $PC_{71}BM/Ni_{80}Fe_{20}$ interface) (**f**), where black and red lines represent spin-up and spin-down electrons, respectively.

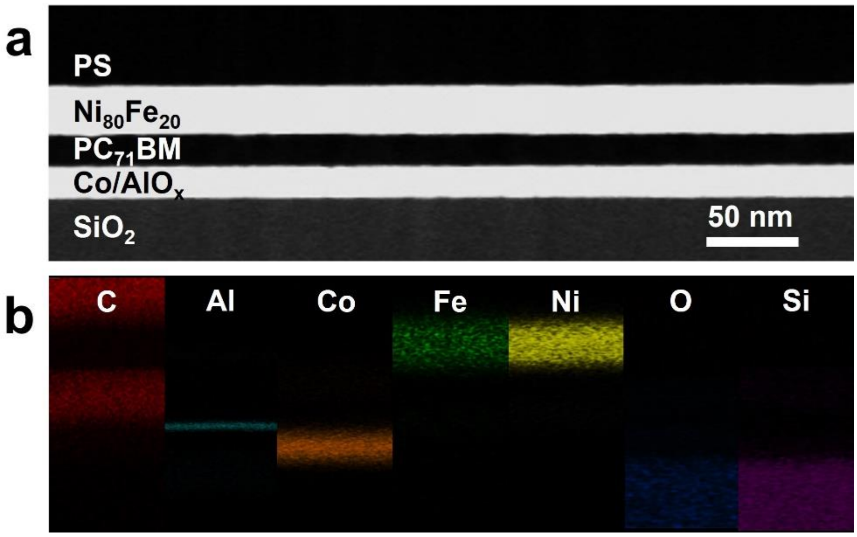

Supplementary Fig. 12

a,**b**, TEM image (**a**) and energy-dispersive X-ray mapping (**b**) of the $PC_{71}BM$-based SV prepared via the transfer technique.

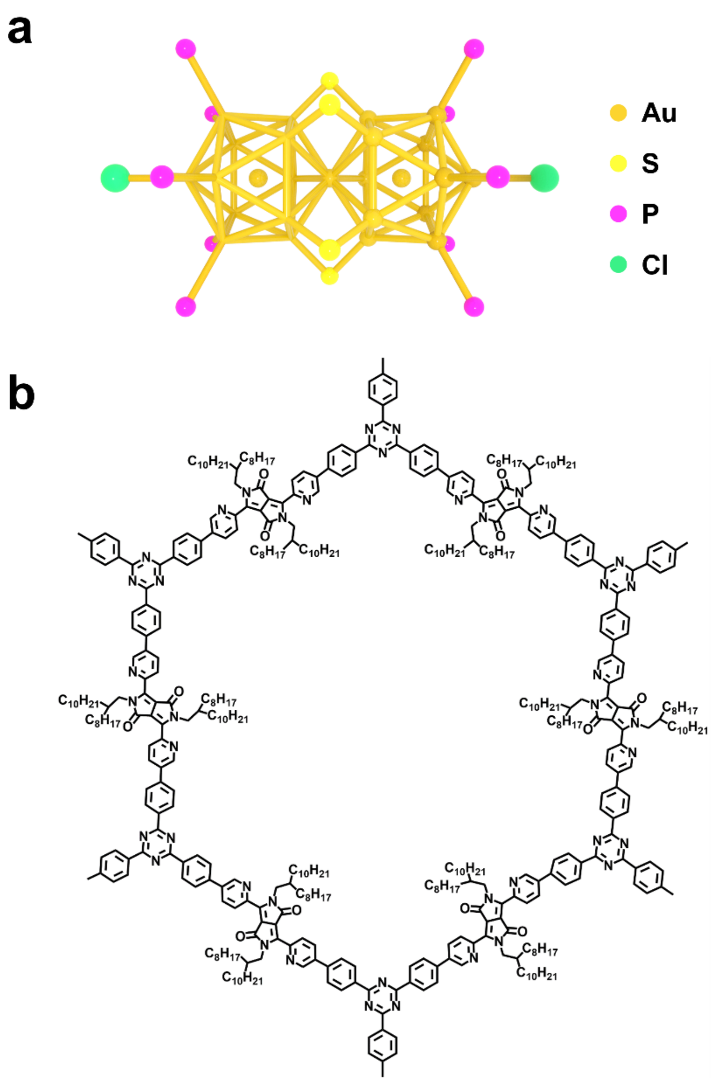

Supplementary Fig. 13

a,**b**, Chemical structures of (**a**) Au_{25} nanoclusters and (**b**) COF employed in this study.

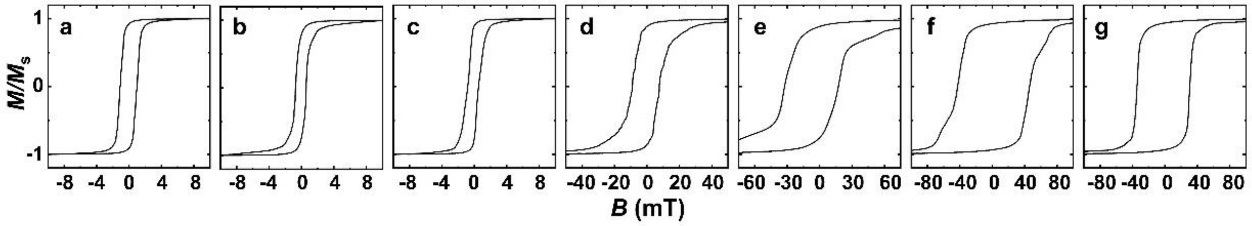

Supplementary Fig. 14

a-g, Hysteresis loops of 5-nm-thick $Ni_{80}Fe_{20}$ on different channel materials, including $[Au_{25}(PPh_3)_{10}(PET)_5Cl_2]^{2+}$ NCs (**a**), lithium fluoride **(b**), spin-coated TPTDPP COF (**c**), P3HT (**d**), CuPc (**e**), tips-pentacene (**f**), in-situ grown TPTDPP COF (**g**).

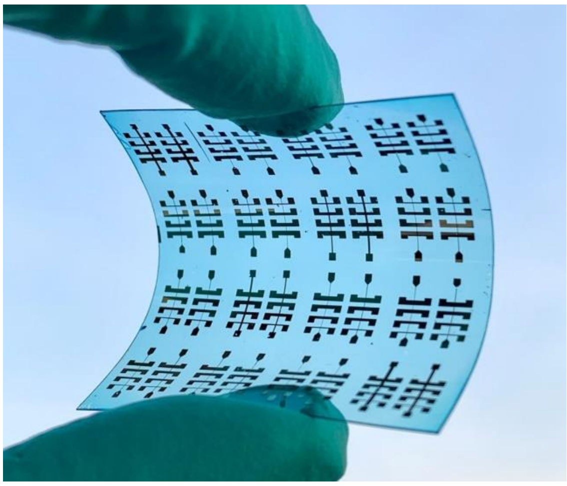

Supplementary Fig. 15

Photo of large-area CuPc-based SV arrays based on flexible PET substrate (4 × 4 cm^2) and processed via transfer technique.

Supplementary References

1. Hou, L. *et al.* Synthesis of a monolayer fullerene network. *Nature* **606**, 507-510 (2022).

2. Hou, J. G., Li, X., Li, Y. & Wang, H. Synthesis and characterization of metal-C_{60} nanostructured films. *Adv. Mater.* **11**, 1124-1126 (1999).
